# Incidence and survival of neuroendocrine neoplasia in England 1995–2018: A retrospective, population-based study

**DOI:** 10.1016/j.lanepe.2022.100510

**Published:** 2022-09-23

**Authors:** Benjamin E. White, Brian Rous, Kandiah Chandrakumaran, Kwok Wong, Catherine Bouvier, Mieke Van Hemelrijck, Gincy George, Beth Russell, Rajaventhan Srirajaskanthan, John K. Ramage

**Affiliations:** a Basingstoke and North Hampshire Hospital, Hampshire Hospitals NHS Foundation Trust, Basingstoke, RG24 9NA, United Kingdom; b King's Health Partners ENETS Center of Excellence, King's College Hospital, London, SE1 9RT, United Kingdom; c Neuroendocrine Cancer UK, Holly House, Leamington Spa, CV32 4JL, United Kingdom; d NHS Digital, 7 and 8 Wellington Place, Leeds, West Yorkshire, LS1 4AP, United Kingdom; e Translational Oncology and Urology Research, School of Cancer & Pharmaceutical Sciences, King's College London, United Kingdom

**Keywords:** Neuroendocrine tumour, Neuroendocrine neoplasia, Carcinoid, Epidemiology, Survival, Incidence, Predictors of survival

## Abstract

**Background:**

Neuroendocrine neoplasia (NEN) incidence is rising internationally. We aimed to evaluate the epidemiology of NEN in England and examine changes in survival over time.

**Methods:**

A retrospective, population-based study using nationally representative data between 1995 and 2018 from the National Cancer Registry and Analysis Service (NCRAS) in England was conducted on 63,949 tumours. Age-standardized incidence was calculated using Office for National Statistics (ONS) data. Overall survival (OS) was calculated using the Kaplan-Meier estimator. Multivariable analysis was performed using an accelerated failure time model.

**Findings:**

Of 63,949 cases, 50.5% (32,309) were female. Age-adjusted incidence increased 3.7-fold between 1995 and 2018 from 2.35 to 8.61 per 100,000. In 2018, highest incidence occurred in lung (1.47 per 100,000), small intestine (1.46 per 100,000), pancreas (1.00 per 100,000) and appendix (0.95 per 100,000). In multivariable analysis, age, sex, morphology, stage, site and deprivation were independent predictors of survival (*p* < 0.001). Survival of the entire cohort, and by primary site, is improving over time.

**Interpretation:**

NEN incidence continues to rise in England with survival improving over time. Relatively high survival compared to other cancers is an issue for long-term outcomes and funding of care.

**Funding:**

Data were extracted and transferred using a grant from Neuroendocrine cancer UK.

**Research in context**

**Evidence before this study**

We searched PubMed for articles published up to the 1st of July 2022 containing the following terms: ("neuroendocrine neoplasia" OR "neuroendocrine tumor" OR "neuroendocrine tumour") AND "incidence" AND "survival". The incidence of neuroendocrine neoplasia (NEN) has been increasing sharply across Europe and the rest of the world since the 2000s, with recently published work suggesting a rate as high as 9 per 100,000 inhabitants. This means NEN can now no longer be defined as a rare cancer. Survival estimates and predictors of survival have not been internationally consistent. Late-stage disease survival tends to be longer than for other solid organ cancers of similar stage contributing to a large number of people now living with NEN. Accurate epidemiological and survival analysis plays a key role in planning for and managing these tumours at the individual and population level, and in communicating with patients. The data source used in the study was the National Cancer Registry and Analysis Service (NCRAS) which records more than 99% of tumours diagnosed in England's National Health Service (NHS).

**Added value of this study**

This comprehensive whole population analysis of NEN is to our knowledge the largest in Europe to date. Age, sex, stage, deprivation, site and morphology were identified as statistically significant independent predictors of survival. Survival is improving over time which might be explained by stage breakdown at each site. There is a survival advantage for females with NEN when compared with males. Identification of poorly differentiated neuroendocrine carcinomas is important due to their poor prognosis compared to neuroendocrine tumours.

**Implications of all the available evidence**

Rising incidence of NEN, coupled with improved survival, means that the cohort of patients living with this disease across Europe is expanding. Improved, tailored commissioning for NEN services is needed to ease the burden on national healthcare systems and chiefly to provide better care for patients living with this condition.

Alt-text: Unlabelled box

## Introduction

Neuroendocrine neoplasia (NEN) is a formerly rare type of cancer found most commonly in the bronchopulmonary and gastrointestinal systems becoming increasingly common over the last decade. The incidence of NEN has been published in registry studies worldwide.^1^^,^^2^ The largest of these registries is the National Cancer Institute's Surveillance, Epidemiology, and End Results (SEER) Program which includes around 30% of the North American population.^3^^,^^4^ Registry data with a significant number of NEN have been analysed from Australia, Norway, Canada, Taiwan, Korea, Germany, France, and other European countries.^5^, ^6^, ^7^, ^8^, ^9^, ^10^, ^11^, ^12^, ^13^, ^14^

There are very few comprehensive studies which include the entirety of a country's population. Differing infrastructure of health care and reporting systems results in varied completeness and accuracy of national databases. Because NEN remains relatively rare, countries with smaller populations can only report on small numbers of tumours. Single-centre, clinical cohorts of NEN are informative but not comprehensive, containing inherent bias as only local patients are included.

Accurate classification of NEN has been challenging due to changing international classification systems. However, a more unified approach is now being used. A precise understanding of incidence and survival for NEN is crucial both to improve clinical outcomes and guide healthcare resource allocation.^2^ Early diagnosis of NEN is related to reduced morbidity and mortality, however patients frequently experience misdiagnosis and significant delays to diagnosis.^15^ Additionally, clinicians, researchers and patients have found it difficult to access information in the same way they might for other cancers.^16^

Due to longer survival than in other cancers, there are many more living with NEN than with other more well-recognised upper gastrointestinal cancers such as pancreatic adenocarcinoma.^17^ Multiple studies show that NEN incidence is rising internationally.^3^^,^^5^^,^^7^^,^^10^^,^^17^ The cause is yet unknown but increased detection seems likely, as well as a possible real increased incidence. A comprehensive Swedish autopsy study comparing post-mortem to real-time diagnosis over a time period showed a high number of undiagnosed NEN in the small bowel.^18^ A Canadian study showed that, despite overall increased NEN incidence, the proportion of those presenting with metastatic disease had decreased significantly, with earlier stage at diagnosis, and posited increased detection as the cause.^8^

Survival for NEN is increasing over time according to other cohorts.^3^ Low mortality rates with increasing incidence may support a hypothesis of increased early detection of tumours.^5^ Increased survival may be related to greater availability of treatment in the form of advances in surgery, radiology, and systemic therapies.^19^ Diagnosing NEN at an earlier stage improves prognosis, and may be important in screening for other solid organ cancers.^18^

This analysis aims to characterize the epidemiology of NEN in England between 1995–2018 and report survival outcomes.

## Method

### Data source

This work utilised data from the National Cancer Registry and Analysis Service (NCRAS) of England, which captures over 99% of tumours recorded in England's National Health Service.^20^^,^^21^ The NCRAS database is updated as histopathological classification systems change, which presents challenges in a rapidly evolving field such as NEN. NCRAS collects stage according to the European Neuroendocrine Tumor Society (ENETS) system for foregut^22^ and mid- and hindgut^23^ tumours and uses the Union for International Cancer Control tumour, node and metastasis system (UICC TNM)^24^ for other sites. Data were analysed on individuals over 16 years of age diagnosed with a NEN between 1995–2018.

### NEN classification and analytic process

NEN occurring between 1995 and 2018 at all anatomical sites between C00 and C80, malignant neoplasms of all sites excluding haematological malignancy, according to the 10th edition of the WHO International Classification of Disease (ICD-10) were included. Morphology codes included 8013 (excluding lung [C34 and C78]), 8041–8045 (excluding lung), 8150–8158, 8240–8247, 8249 and 9091 according to the WHO International Classification of Diseases for Oncology, 3rd Edition (ICD-O-3).^25^ This was consistent with previously published work on NEN using NCRAS data.^17^ See Supplementary Table 1 for full ICD descriptions. Large cell neuroendocrine and small cell carcinomas of the lung were excluded as the high incidence in this organ would skew the results. Goblet cell adenocarcinoma (GCA) (ICD-O-3: 8243) were excluded from the dataset in view of their reclassification as non-NEN.^26^ Duplicates tumours and tumours recorded as “death certificate only”, which made up less than 0.1% of tumours, were excluded.^27^^,^^28^

**Figure 1:**
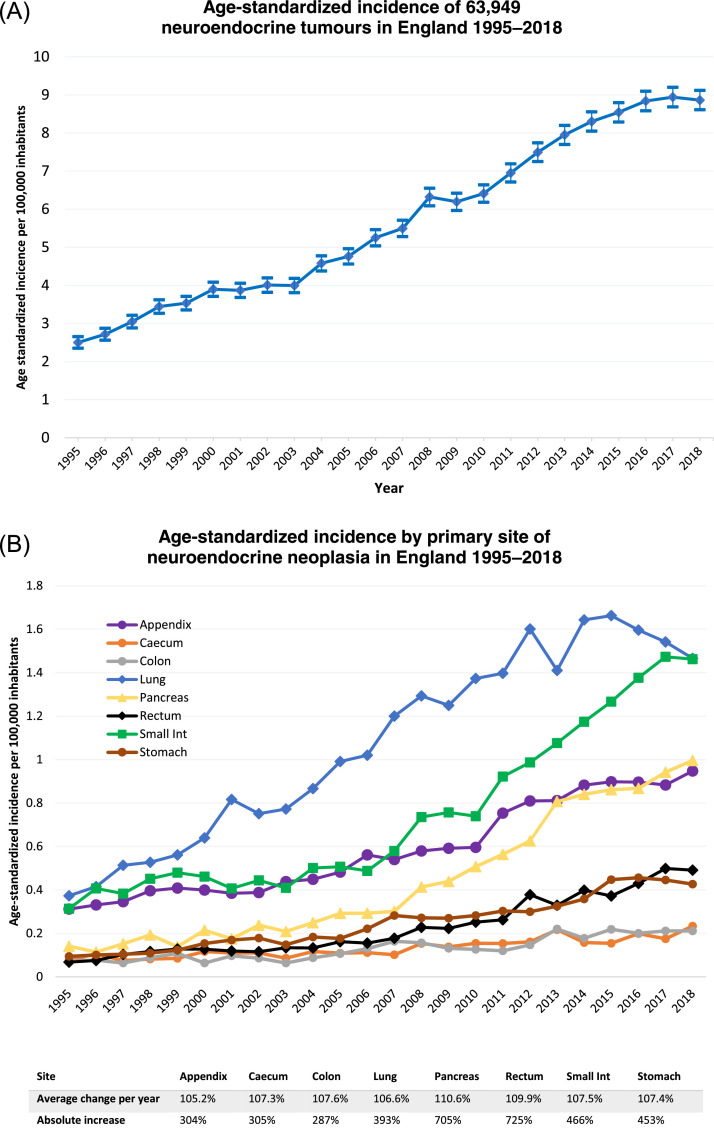
(A) Age standardized incidence of 63,949 neuroendocrine neoplasia from 1995–2018 in England. 95% confidence interval displayed. Data source: NCRAS. (B) Age standardized incidence of 40,534 NEN at main sites from 1995–2018 in England with average percentage change per year and absolute rise. Data source: NCRAS.

Mixed Neuroendocrine Non-Neuroendocrine Neoplasms (MiNEN) (formerly termed mixed adenoneuroendocrine carcinomas (MANEC)) were included in overall age standardized incidence analysis (Figure 1 and Table 1) between 1995–2018 but excluded from the further 2012–2018 survival analysis group due to recent debate about their status as ‘true’ NEN. Merkel cell tumours, although classified as NEN, were excluded from 2012–2018 survival estimates as they are now seen as a distinct group. Post-2012 tumours were chosen for the main survival analysis due to markedly improved coding and classification in recent years, although unclassified stage (25.6%) were excluded.

**Table 1:** (A) Demographics of 63,949 NEN. (B) Age and sex distribution of 40,534 NEN between 1995–2018.

Cohort Demographics									
(A)									
		1995–2002		2003–2010		2011–2018		Total	
**Age (Years, Median (IQR))**		66 (53–75)		67 (55–76)		67 (55–76)		67 (55–76)	
		***n***	%	***n***	%	***n***	%	***n***	%
**Sex**	Male	5562	48.4%	9628	49.5%	16450	49.8%	31640	49.48%
	Female	5921	51.6%	9813	50.5%	16575	50.2%	32309	50.52%
**Ethnicity**	Asian	123	1.1%	457	2.4%	973	2.9%	1553	2.43%
	Black	95	0.8%	278	1.4%	644	2.0%	1017	1.59%
	Mixed race	16	0.1%	74	0.4%	142	0.4%	232	0.36%
	Other	65	0.6%	144	0.7%	370	1.1%	579	0.91%
	White	7274	63.3%	17538	90.2%	29486	89.3%	54298	84.91%
	Unknown	3910	34.1%	950	4.9%	1410	4.3%	6270	9.80%
**Site**	Appendix	1424	12.4%	2123	10.9%	3682	11.1%	7229	11.30%
	Caecum	309	2.7%	428	2.2%	717	2.2%	1454	2.27%
	Colon	268	2.3%	424	2.2%	745	2.3%	1437	2.25%
	Lung	1967	17.1%	3937	20.3%	6120	18.5%	12024	18.80%
	Other	4748	41.3%	7674	39.5%	10471	31.7%	22893	35.80%
	Pancreas	591	5.1%	1245	6.4%	3284	9.9%	5120	8.01%
	Rectum	365	3.2%	678	3.5%	1608	4.9%	2651	4.15%
	Small intestine	1380	12.0%	2111	10.9%	4867	14.7%	8358	13.07%
	Stomach	431	3.8%	821	4.2%	1531	4.6%	2783	4.35%
**Stage**	Stage 1	50	0.4%	293	1.5%	6136	18.6%	6479	10.13%
	Stage 2	42	0.4%	206	1.1%	3195	9.7%	3443	5.38%
	Stage 3	65	0.6%	302	1.6%	3792	11.5%	4159	6.50%
	Stage 4	149	1.3%	1250	6.4%	7300	22.1%	8699	13.60%
	Unclassified	11177	97.3%	17390	89.5%	12602	38.2%	41169	64.38%
**IMD**	1 – Least deprived	2096	18.3%	3766	19.4%	6769	20.5%	12631	19.75%
	2	2414	21.0%	4087	21.0%	7062	21.4%	13563	21.21%
	3	2324	20.2%	4076	21.0%	6957	21.1%	13357	20.89%
	4	2339	20.4%	3834	19.7%	6198	18.8%	12371	19.35%
	5 – Most deprived	2310	20.1%	3678	18.9%	6039	18.3%	12027	18.81%

(B)			
Site	N	Median age (IQR)	Males %
Appendix	7031	39 (25–58)	39.3%
Caecum	1411	68 (59–75)	45.0%
Colon	1387	69 (59–77)	57.2%
Lung	11930	66 (56–74)	44.6%
Pancreas	5079	63 (52–72)	54.2%
Rectum	2622	61 (50–71)	55.7%
Small intestine	8335	68 (59–76)	56.5%
Stomach	2739	69 (58–77)	52.1%

Site groups were created from histological codes. Main sites were defined as appendix, caecum, colon, lung, pancreas, rectum, small intestine or stomach, in line with other series.^3^

Although all tumours have a histopathological classification, Ki-67 index is not yet available, so we therefore grouped NENs by morphology either as well-differentiated neuroendocrine tumours (NET) or poorly-differentiated neuroendocrine carcinomas (NEC) in line with other recently published work.^29^ Tumours counted as NETs included carcinoids of typical, atypical, tubular, and other well differentiated neoplasms such as insulinoma and glucagonoma. NECs included all carcinomas and tumours with large and small cell neuroendocrine differentiation.

Variables suitable to be included in the analysis were site, age group, sex, Index of multiple deprivation’ (IMD), morphology and stage. IMD is a measure of relative deprivation for small areas of England (lower layer super output areas) and is calculated using seven domains with relative weights: income (22.5%), employment (22.5), education (13.5%), health (13.5%), crime (9.3%), housing (9.3%) and environment (9.3%). The following other variables were excluded due to a significant amount of incomplete or missing data: Charlson comorbidity index, route to diagnosis, chemotherapy regimens and tumour size. Ethnicity was excluded from multivariable analysis due to a large weighting (89%) toward white people in England making results for comparison with other ethnicities unreliable.

For comparison over time, NEN was split into three equal year groups 1995–2002, 2003–2010 and 2011–2018 and compared by site using the K-M estimator. Other variables could not be used due to missing data. Overall survival was used rather than cancer-specific (net) survival in view of concerns about the reliability of coding cancer-related deaths in NEN and because of the risk of missing cancer-related deaths, with NEN tending to have effects on multiple organ systems.

### Statistical analysis

Categorical variables were presented with percentage and continuous variables reported with median and interquartile range (IQR). Age standardized incidence was calculated with the ONS method using the European standard population 2013. The primary end point was overall survival (OS), calculated from the date of diagnosis and censored on March 31 2020, estimated with the Kaplan-Meier estimator and given with the 95% Confidence Interval (95% CI). A p-value less than 0.05 was deemed significant. Cox regression multivariable analysis included sex, morphology, age group, stage, site and deprivation. Of these variables, only sex and deprivation met proportional hazards assumptions, the rest were included in the final multivariable model as time-varying covariates (TVC). Accelerated Failure Time (AFT) models were tested for significance against the null models (Cox) using a likelihood ratio test. Statistical analyses and plots were performed using SPSS version 27 (IBM Corp., Armonk, N.Y., USA) and STATA/MP 16.0 (College Station, TX: StataCorp LLC).

### Role of the funding source

A grant for £10,000 was awarded by Neuroendocrine Cancer UK (NCUK) in order to handle prepation, processing, secure transfer of data and follow-up support from NCRAS/NHS Digital to BEW/Hampshire Hospitals NHS Foundation Trust. NCUK provided a link to input on patient and public involvement for the research team. NCUK had no influence on the limit of analysis or published results. As a co-author, CB, Chief Executive Officer of NCUK, advised on patient and public involvement throughout and provided review input to the research.

## Results

A total of 63,949 NEN were registered on the NCRAS database in England between 1995 and 2018. As of March 31st, 2020, there were 26,607 people in England living with NEN diagnosed between 1995–2018. Patient characteristics for the cohort divided into three equal time periods, 1995–2002, 2003–2010 and 2011–2018 are given in Table 1A. For the demographics table divided by NET versus NEC, please see Supplementary Table 2A and B.

The incidence of NEN has risen steadily over the 24–year period. As demonstrated in Figure 1, incidence of NEN in England was 8.8 per 100,000 inhabitants in 2018, increasing from 2.5 per 100,000 in 1995, an absolute increase of 371% during the time period. Incidence at every main primary site of NEN rose yearly, with pancreatic and rectal NEN seeing the highest yearly average increases (110.6% and 109.9% per year respectively). Small intestinal and pancreatic NEN have increased markedly compared to other sites over the last ten years of the time period, whilst lung NEN appears to be tailing off (Figure 1B).

The median age of the cohort was 67 (IQR 55–76). As shown in Table 1B, the median age at all main sites was between 60–70 years, except appendix, where the median age was 39 years (IQR 25–58). Of the main sites, lung (n=11,930) and small intestine (n=8335) were the most numerous. Sex was predominantly male in colonic, pancreatic, rectal, small intestinal and gastric NEN and predominantly female in appendiceal and lung (Table 1B).

There were 14,834 staged tumours between 2012-2018 which classified morphologically as NET or NEC. Patient ethnicity (England 2011 census in parentheses) was 89.0% (83%) White, 2.3% (3%) Black and 2.9% (7%) Asian. Proportion of NEC and proportion of advanced stage increased with age (supplementary Figure 1).

### Survival analysis

**Figure 2:**
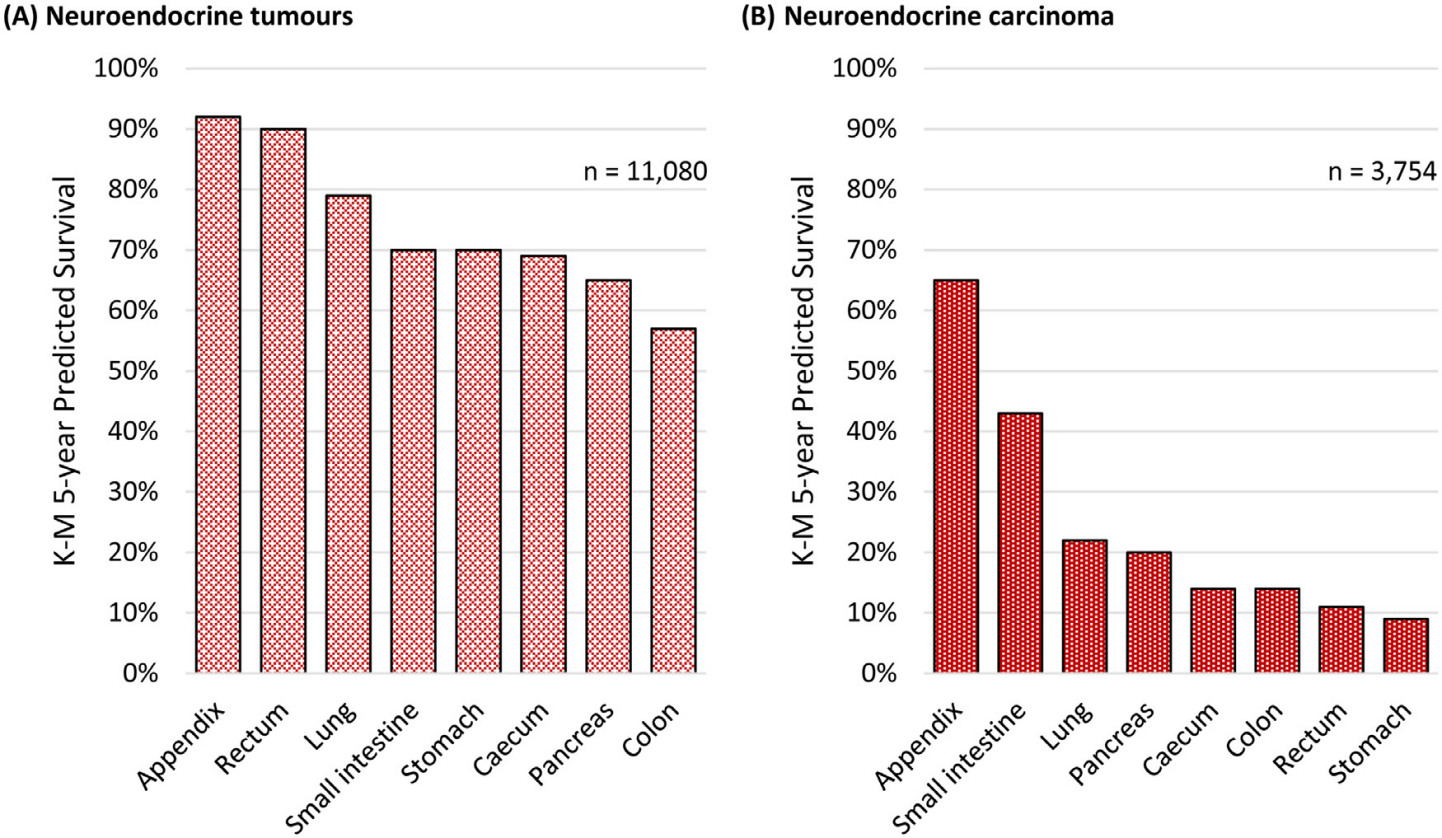
Kaplan–Meier predicted 5–year survival of (A) 11,080 neuroendocrine tumours and (B) 3,754 neuroendocrine carcinomas between 2012 and 2018 in England. Source data: NCRAS.

In total 14,834 tumours registered between 2012 and 2018 were suitable for the primary survival analysis. Five-year survival for the main sites is displayed in Figure 2 (A) and (B). Appendix had the best five-year survival of any main site whether the tumour was a NET (92%) or a NEC (65%). Rectal NET had one of the highest five-year survivals (90%), however rectal NEC had almost the worst (11%). Small intestinal and appendiceal NEC had substantially better five-year survival compared to NEC at all other main sites (43–65% small intestine & appendix versus 9–22% all other sites). We have specified the proportion of NET versus NEC at each main site, by stage, in Supplementary Table 3. In Supplementary Table 4, five-year survival by site and stage are shown as a reference guide for clinicians (this table does not account for age).

**Figure 3:**
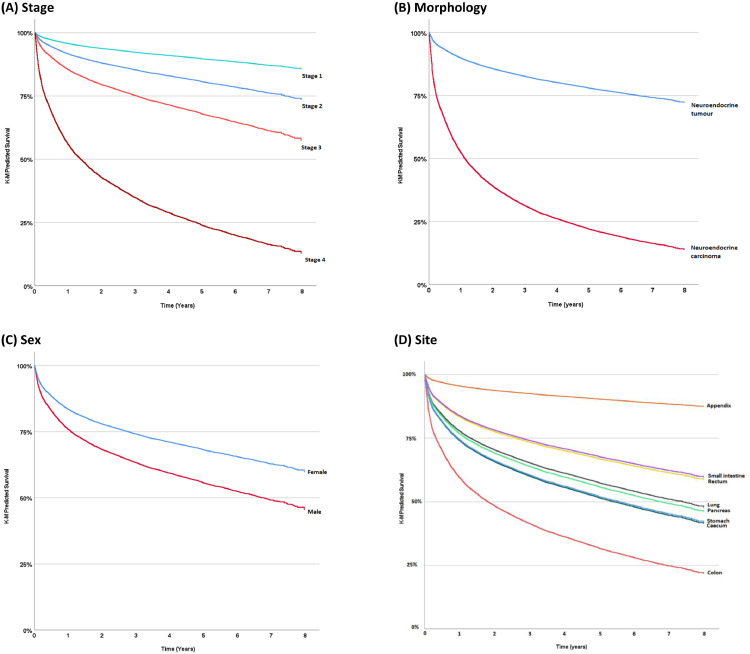
Kaplan-Meier predicted survival of NEN by stage (A), tumour morphology (B), sex (C) and site (D) from 2012–2018 in England. Source data: NCRAS.

Proportional hazards testing of each variable revealed hazards remained constant for sex and IMD. Time-varying covariates were used for the others. In multivariable analysis, the AFT model was significant at *p* < 0.05 and an improvement over the original Cox regression model using a likelihood ratio test (*p* < 0.001). Age group, sex, morphology (in terms of NET vs NEC), stage and deprivation were independent predictors of survival (*p* < 0.001 except site *p* = 0.041) (Table 2). Being over 75 years conferred the highest hazard ratio (HR) (HR = 7.72 (95% CI: 5.06–11.80)) as compared to under 30 years. This was followed by stage 4 disease (HR = 2.11 (95% CI: 2.01–2.23)) as compared to stage 1. In the model, male patients were 43% more likely to die than female patients (HR = 1.43 (95% CI: 1.35–1.51). A person with a NEC (HR = 1.29 (95% CI: 1.25–1.33)) had a 29% greater risk of death than a NET. Those in the most deprived quintile were 32% more likely to die than those in the least deprived (HR = 1.32 (95% CI: 1.22–1.45). Figure 3 shows Kaplan-Meier Predicted survival plots for stage (A), tumour morphology (B), sex (C) and site (D).

**Table 2:** Hazard ratios calculated with multivariable analysis using Cox Regression and an accelerated failure time model. LCI = 95% confidence interval lower bound, UCI = 95% confidence interval upper bound *denotes covariate with time-varying effect.

Multivariable analysis of survival					
		HR	LCI	UCI	Sig.
Age group*					<0.001
	≥ 29	1			
	30–54	4.41	2.88	6.74	
	55–64	5.35	3.50	8.18	
	65–74	6.13	4.01	9.37	
	75+	7.72	5.06	11.80	
					
**Sex**					<0.001
	Female	1			
	Male	1.43	1.35	1.51	
					
Morphology*					<0.001
	NET	1			
	NEC	1.29	1.25	1.33	
					
Stage*					<0.001
	1	1			
	2	1.38	1.29	1.46	
	3	1.58	1.49	1.68	
	4	2.11	2.01	2.23	
					
Site of tumour*					0.041
	Appendix	1			
	Caecum	1.00	0.90	1.11	
	Colon	1.14	1.04	1.25	
	Lung	1.22	1.13	1.31	
	Pancreas	1.18	1.09	1.28	
	Rectum	1.27	1.15	1.41	
	Small intestine	0.94	0.87	1.02	
	Stomach	1.26	1.14	1.33	
					
**IMD**					<0.001
	1 - least deprived	1			
	2	1.11	1.02	1.21	
	3	1.09	1.00	1.19	
	4	1.21	1.11	1.33	
	5 - most deprived	1.32	1.22	1.45

Survival improved over time at all sites between 1995–2018, as shown in Supplementary Figure 2. The largest increase in five-year survival over time was seen in NEN of the small intestine (from 7% to 52%), colon (4–28%) and pancreas (7–45%). The smallest increase was seen in appendiceal (58–87%), lung (19–48%) and rectal (25–63%) NEN.

## Discussion

To our knowledge this analysis of 63,949 NEN in England is the largest complete single country population analysis of NEN published to date. Incidence of NEN in England rose by 371% between 1995 and 2018 but in the same time period all-cancer incidence (excluding non-melanoma skin cell cancer) rose by only 116%.^30^ We demonstrated a progressive rise in incidence over nearly three decades, however, the cause for the rise is not clear. It is likely to be a combination of improved histological classification and recording, increased detection rate possibly combined with an increase in actual tumour development.

The median age of the primary sites of NEN was 61–69 except in appendix where it was 39. This is consistent with other studies and is likely a result of appendix NEN found incidentally during or after appendicectomy.^31^ Highest absolute increases of incidence of GEP-NEN from 1995–2018 occurred in the rectum, pancreas and small intestine, the same three sites as in SEER analysis.^32^ The most common primary site of GEP-NEN were the small intestine, pancreas and appendix, differing from SEER where small intestine, rectal and pancreas were most common.^3^ It is not clear why there are currently less rectal NEN seen in our data but screening programmes may play a role, as well as differences in ethnic makeup of the nations.^3^

NEN of the small intestine and pancreas have seen the most marked increase in number over time yet lung NEN have seen a plateau and decrease in recent years, again. The cause of these changes is not clear but refinement of the classification systems may be playing a role.

A Canadian study suggested that increased detection was the cause of apparent increased incidence by demonstrating that the proportion of metastatic presentation was decreasing.^8^ Incidence of small intestine neuroendocrine tumours increased 5-fold on examining an autopsy registry in Malmo.^18^ There are however marked differences by site according to different countries, with some countries seeing a decrease in certain subsets.^10^

We have demonstrated that the classification of NEN as either NET or a NEC, with appropriate staging, is very important, giving the clinician to a better idea of prognosis (Supplementary Table 1). Histology of NECs showed 30% had small cell differentiation which is similar to the 34% observed in North America.^3^ Studies including NEC have been published less commonly and in far less number than NET due to classification issues and rarity^2^ but this study provides a large number of comparable NEC. In general, five-year survival of a stage 4 NEC was much worse than a stage 4 NET at the same site, for example the stomach (NEC 2% vs. NET 21%), the exception being the small intestine, where five-year survival of a stage 4 tumour was comparable (NEC 32% vs. NET 43%).

Survival was better in females compared to males, this has been described in other recent series.^5^^,^^10^^,^^33^ We have shown significant differences in incidence and survival by sex, reflected in other registry studies, and this merits further investigation. There is no cause known for these differences, however it is thought that a combination of biology and behaviour may contribute.^34^, ^35^ The most deprived experienced the worst survival, similar to other cancers.^36^

We showed how survival over time has changed by main site of primary tumour (Supplementary Figure 2). Marked increases over time in five-year survival were seen in NEN of the small intestine (from 7% to 52%) and pancreas (7–45%). It is possible that there is a mixed effect occurring in this improvement over time: where sites are mainly early stage tumours eg. appendix and lung, increased detection could be playing a role, however in all other main sites where late stage tumours predominate, it could be due to improved systemic treatment. We have also shown 1-, 3-, and 5-year survival by site of primary, stage and morphology (Supplementary Table 1). In addition, we have shown that sex, ethnicity, deprivation, stage and grade of tumour have statistically significant hazard ratios affecting survival (Table 2). We have demonstrated that increasing stage correlates closely with worsening survival, and age at diagnosis is a dominant factor for outcomes. The relationship between age and other factors such as comorbidity or concomitant medication could be explored in the future. We plan to explore the relationship between NEN and metachronous tumours using standardized mortality ratios (SMRs) in another concurrent body of work.

The North American SEER data shows no improvement in survival over time in higher stage disease, but some improvement in lower stage disease. Although our cohort is not directly comparable due to differing classification systems and completeness of data, we did observe survival increasing over time. In SEER analyses, likely due to increasingly accurate imaging including PET, stage migration (increasing stage from 3 to 4) has been seen over time. In contrast to SEER, in a Canadian study,^8^ the proportion of patients with higher stage in fact decreased over time, with the authors of this study concluding increased detection might explain increased incidence over time. A Norwegian population-based study of 2030 NENs from 1993–2004 showed similar incidence to SEER with similar sex and age differences,^6^ and similar findings from Norway were reported in 2016.^7^ The changes in stage and grade over time have therefore been inconsistent in different countries, and this in part may represent changes in measurement methods and in classification systems.

Similar population databases have shown incidence and survival data in other countries. The SEER data from the USA has been widely reported^3^^,^^37^^,^^38^ with similar numbers, but this database only covers around a third of the US population. Missing data on grade and stage was similar and stage was not reported by WHO criteria, and a recent update only includes data to 2014.^4^ The latter study includes Goblet Cell carcinoma (GCC), adenocarcinoid and mixed adeno with NEN (MiNEN) in the main analysis. We excluded these on the basis that GCC and adenocarcinoid are no longer considered part of the NEN spectrum, and that MiNEN are more likely to behave like adenocarcinoma.

Inequalities in cancer outcomes is something widely known and is failing to improve in England.^36^ We demonstrate significantly worse survival in the most deprived quintile compared to the least deprived, with the most deprived being 32% more likely to die. This suggests that, as in other conditions, resources should be focused on those in the most deprived areas.

Whilst this is the largest study of NEN for an entire country's population, there are some limitations. Missing data for grade and to a lesser extent stage impacted analysis, although stage data 70% complete in the most recent years of the 2012-2018 subset used for survival analysis. There is no Ki-67 available for analysis yet, and therefore we attempted to compensate for missing grade by using morphology as a surrogate. Five-year survival over time could only be reliably compared using site of primary due to historic lack of recorded stage and changing interpretation of morphology. It is not yet possible within the dataset to use other linked clinical variables known to be relevant in cancer such as alcohol, smoking or obesity. A limitation of all studies examining NEN over long time periods is changes in histopathological classification, however, survival estimates for the main group analysis were taken from 2012 onwards to enable the highest accuracy. Limitations of a retrospective population-based approach to research also apply.

NENs are an under-recognised malignancy with an increasing health care burden. Indeed, according to our data, NEN is now the 10^th^ most prevalent cancer in England, and the most prevalent gastrointestinal malignancy after colorectal cancer.^39^ This data on survival is useful for clinicians and health care professionals looking at prognosis of NEN.

In summary, our results from a large population linkage analysis of NEN in England show a significant increase in incidence of NEN, which is consistent with data from other countries. Increasing health care resources are being utilised in NEN due to prolonged survival compared to many other malignancies. Classification of NEN as a NET or a NEC has clear prognostic potential. This work has demonstrated a worse outcome for those from deprived areas and lower socioeconomic status. These findings may aid clinical decision-making and help inform patients about their diagnosis. Future analysis may be widened to include all UK population and also include Ki-67 classification for more accurate WHO grading.

## Contributors

B.E.W. was the author of the original and final draft. All authors contributed to reviewing and editing of drafts equally. Below details authors’ specific contributions.

B.E.W.: Conceptualization; Data curation; Formal analysis; Funding acquisition; Investigation; Methodology; Project administration; Resources; Software; Validation; Visualization

B.R.: Data curation; Investigation; Methodology; Supervision; Validation; Visualization

K.C.: Data curation; Formal analysis; Investigation; Methodology; Supervision; Validation

K.W.: Data curation; Formal analysis; Investigation; Methodology; Supervision; Validation

C.B.: Conceptualization; Funding acquisition; Investigation; Resources; Visualization

M.V.H.: Formal analysis; Investigation; Methodology; Supervision; Validation; Visualization

G.G.: Formal analysis; Investigation; Methodology; Validation

B.R.: Formal analysis; Investigation; Methodology; Validation

R.S.: Conceptualization; Funding acquisition; Investigation; Methodology; Project administration; Resources; Supervision; Validation; Visualization

J.K.R.: Conceptualization; Formal analysis; Funding acquisition; Investigation; Methodology; Project administration; Resources; Software; Supervision; Validation; Visualization.

## Data sharing statement

Data cannot be made public due to legal rules of NCRAS database.

## Ethics approval and consent to participate

NHS Health Research Authority REC reference: 20/NW/0342

IRAS project ID: 284875

Sponsor: Hampshire Hospitals NHS Foundation Trust

## Consent for publication

See ethical approval.

## Declaration of interests

JKR supervised securing funding for the grant from Neuroendocrine Cancer UK (NCUK) to process the data in the initial planning of the work. CB states that NCUK receives donation, grant and sponsorship money from patients, businesses and not-for-profit organisations. CB is a board member of the International Neuroendocrine Cancer Alliance (INCA). The other authors declare no conflict of interest.

## Appendix Supplementary materials

Image, application 1

Image, application 2

Image, application 3

Image, application 4

Image, application 5

Image, application 6

Image, application 7
