# Supplementary material for: Incidence and survival of neuroendocrine neoplasia in England 1995–2018: A retrospective, population-based study
Source: Lancet Reg Health Eur. 2022 Sep 23;23:100510. doi: 10.1016/j.lanepe.2022.100510 (PMC9513765; doi:10.1016/j.lanepe.2022.100510)
Supplement: Supplementary file 1 [file mmc1.pdf]

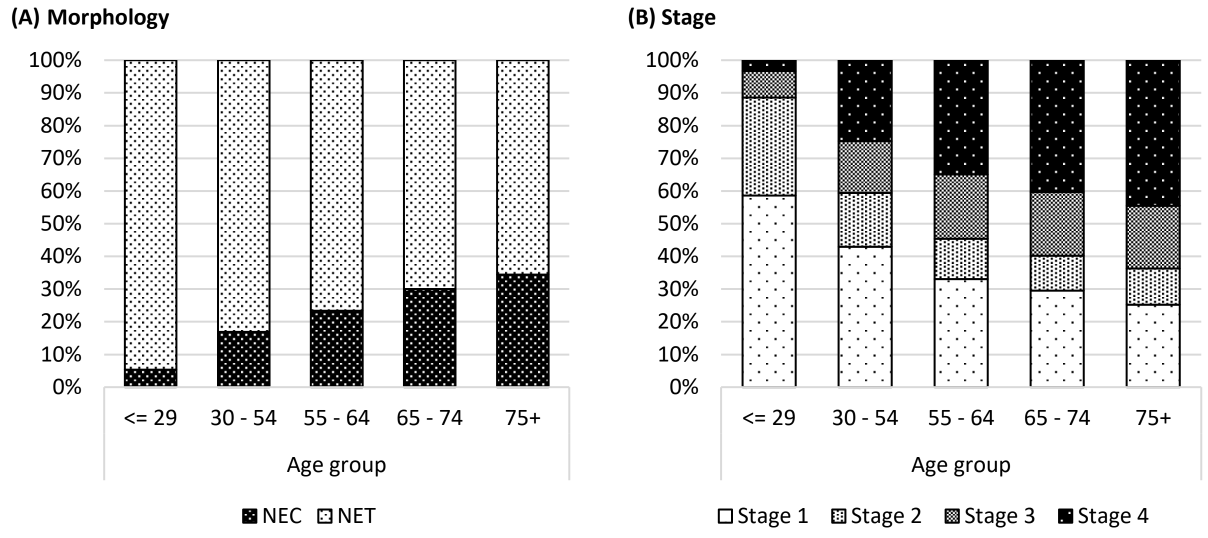

Supplementary Figure 1. Age group stratification of 14,834 neuroendocrine neoplasia between 2012-2018 by (A) morphology and (B) stage.
