# Supplementary material for: Incidence and survival of neuroendocrine neoplasia in England 1995–2018: A retrospective, population-based study
Source: Lancet Reg Health Eur. 2022 Sep 23;23:100510. doi: 10.1016/j.lanepe.2022.100510 (PMC9513765; doi:10.1016/j.lanepe.2022.100510)
Supplement: Supplementary file 2 [file mmc2.pdf]

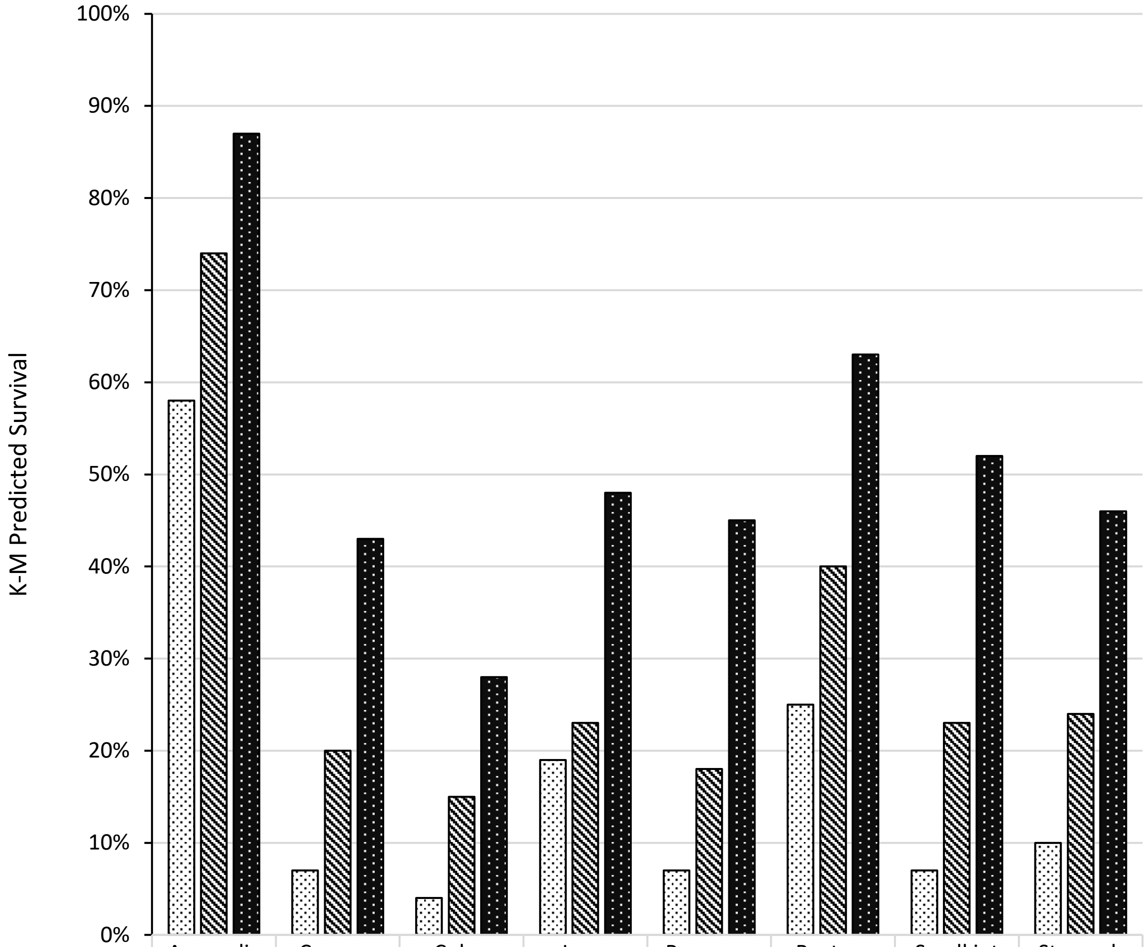

	Appendix	Caecum	Colon	Lung	Pancreas	Rectum	Small int	Stomach
1995-2002	58%	7%	4%	19%	7%	25%	7%	10%
2003-2010	74%	20%	15%	23%	18%	40%	23%	24%
2011-2018	87%	43%	28%	48%	45%	63%	52%	46%

Site

Supplementary Figure 2. Kaplan-Meier predicted 5–year survival of 40,534 neuroendocrine tumours by site over time from 1995–2018 in England.
