# Supplementary material for: Incidence and survival of neuroendocrine neoplasia in England 1995–2018: A retrospective, population-based study
Source: Lancet Reg Health Eur. 2022 Sep 23;23:100510. doi: 10.1016/j.lanepe.2022.100510 (PMC9513765; doi:10.1016/j.lanepe.2022.100510)
Supplement: Supplementary file 3 [file mmc3.docx]

| **ICDO3.2** | **Level** | **Term** |
| --- | --- | --- |
| 8013/3 | Preferred | Large cell neuroendocrine carcinoma |
| 8013/3 | Related | Combined large cell neuroendocrine carcinoma |
| 8041/3 | Preferred | Small cell carcinoma, NOS |
| 8041/3 | Synonym | Reserve cell carcinoma |
| 8041/3 | Synonym | Round cell carcinoma |
| 8041/3 | Related | Small cell neuroendocrine carcinoma |
| 8041/3 | Synonym | Small cell carcinoma, pulmonary type |
| 8042/3 | Preferred | Oat cell carcinoma |
| 8043/3 | Preferred | Small cell carcinoma, fusiform cell |
| 8044/3 | Preferred | Small cell carcinoma, intermediate cell |
| 8044/3 | Related | Small cell carcinoma, hypercalcemic type |
| 8045/3 | Preferred | Combined small cell carcinoma |
| 8045/3 | Synonym | Mixed small cell carcinoma |
| 8045/3 | Related | Combined small cell-adenocarcinoma |
| 8045/3 | Related | Combined small cell-large cell carcinoma |
| 8045/3 | Related | Combined small cell-squamous cell carcinoma |
| 8150/0 | Preferred | Pancreatic neuroendocrine microadenoma |
| 8150/0 | Synonym | Pancreatic endocrine tumor, benign |
| 8150/0 | Synonym | Islet cell tumor, benign |
| 8150/0 | Related | Pancreatic microadenoma |
| 8150/3 | Preferred | Pancreatic neuroendocrine tumor, nonfunctioning |
| 8150/3 | Synonym | Pancreatic endocrine tumor, nonfunctioning |
| 8150/3 | Synonym | Pancreatic endocrine tumor, NOS |
| 8150/3 | Related | Islet cell adenoma |
| 8150/3 | Related | Islet cell adenomatosis |
| 8150/3 | Related | Nesidioblastoma |
| 8150/3 | Related | Islet cell tumor, NOS |
| 8150/3 | Related | Islet cell adenocarcinoma |
| 8150/3 | Related | Islet cell carcinoma |
| 8151/3 | Preferred | Insulinoma, NOS |
| 8151/3 | Synonym | Beta cell adenoma |
| 8151/3 | Synonym | Beta cell tumor |
| 8152/3 | Preferred | Glucagonoma |
| 8152/3 | Synonym | Alpha cell tumor |
| 8152/3 | Related | Enteroglucagonoma |
| 8152/3 | Related | Glucagon-like peptide-producing tumor |
| 8152/3 | Related | L-cell tumor |
| 8152/3 | Related | Pancreatic peptide and pancreatic peptide-like peptide within terminal tyrosine amide producing tumor |
| 8152/3 | Synonym | PP/PYY producing tumor |
| 8153/3 | Preferred | Gastrinoma |
| 8153/3 | Synonym | G cell tumor |
| 8153/3 | Synonym | Gastrin cell tumor |
| 8154/3 | Preferred | Mixed neuroendocrine non-neuroendocrine neoplasm (MiNEN) |
| 8154/3 | Related | Mixed pancreatic endocrine and exocrine tumor, malignant |
| 8154/3 | Synonym | Mixed islet cell and exocrine adenocarcinoma |
| 8154/3 | Related | Mixed acinar-endocrine carcinoma |
| 8154/3 | Synonym | Mixed acinar-neuroendocrine carcinoma |
| 8154/3 | Related | Mixed acinar-endocrine-ductal carcinoma |
| 8154/3 | Related | Mixed ductal-endocrine carcinoma |
| 8154/3 | Synonym | Mixed ductal-neuroendocrine carcinoma |
| 8154/3 | Related | Mixed endocrine and exocrine adenocarcinoma |
| 8155/3 | Preferred | Vipoma |
| 8156/3 | Preferred | Somatostatinoma |
| 8156/3 | Synonym | Somatostatin cell tumor |
| 8158/3 | Preferred | ACTH-producing tumor |
| 8158/3 | Related | Endocrine tumor, functioning, NOS |
| 8240/3 | Preferred | Neuroendocrine tumor, NOS |
| 8240/3 | Synonym | Carcinoid tumor, NOS |
| 8240/3 | Synonym | Carcinoid, NOS |
| 8240/3 | Related | Bronchial adenoma, carcinoid |
| 8240/3 | Synonym | Neuroendocrine carcinoma, low grade |
| 8240/3 | Synonym | Neuroendocrine carcinoma, well differentiated |
| 8240/3 | Related | Neuroendocrine tumor, grade 1 |
| 8240/3 | Synonym | Typical carcinoid |
| 8241/3 | Preferred | Enterochromaffin cell carcinoid |
| 8241/3 | Synonym | Argentaffinoma |
| 8241/3 | Synonym | Carcinoid tumor, argentaffin |
| 8241/3 | Synonym | EC cell carcinoid |
| 8241/3 | Synonym | Serotonin producing carcinoid |
| 8241/3 | Synonym | Serotonin producing tumor |
| 8242/3 | Preferred | Enterochromaffin-like cell tumor |
| 8242/3 | Synonym | ECL cell carcinoid |
| 8243/3 | Preferred | Goblet cell carcinoid |
| 8243/3 | Synonym | Mucinous carcinoid |
| 8243/3 | Synonym | Mucocarcinoid tumor |
| 8244/3 | Preferred | Mixed adenoneuroendocrine carcinoma |
| 8244/3 | Synonym | Combined carcinoid and adenocarcinoma |
| 8244/3 | Synonym | Mixed carcinoid and adenocarcinoma |
| 8244/3 | Synonym | Composite carcinoid |
| 8244/3 | Synonym | MANEC |
| 8244/3 | Synonym | Mixed carcinoid-adenocarcinoma |
| 8245/1 | Preferred | Tubular carcinoid |
| 8245/3 | Preferred | Adenocarcinoid tumor |
| 8246/3 | Preferred | Neuroendocrine carcinoma, NOS |
| 8246/3 | Synonym | Poorly differentiated neuroendocrine neoplasm |
| 8247/3 | Preferred | Merkel cell carcinoma |
| 8247/3 | Synonym | Merkel cell tumor |
| 8247/3 | Synonym | Primary cutaneous neuroendocrine carcinoma |
| 8249/3 | Preferred | Neuroendocrine tumor, grade 2 |
| 8249/3 | Synonym | Atypical carcinoid tumor |
| 8249/3 | Related | Neuroendocrine carcinoma, moderately differentiated |
| 8249/3 | Related | Neuroendocrine tumor, grade 3 |
| 9091/1 | Preferred | Strumal carcinoid |
| 9091/1 | Synonym | Struma ovarii and carcinoid |

**Supplementary Table 1**: Morphology codes
