# Supplementary material for: Incidence and survival of neuroendocrine neoplasia in England 1995–2018: A retrospective, population-based study
Source: Lancet Reg Health Eur. 2022 Sep 23;23:100510. doi: 10.1016/j.lanepe.2022.100510 (PMC9513765; doi:10.1016/j.lanepe.2022.100510)
Supplement: Supplementary file 4 [file mmc4.docx]

|  | | **1995-2002** | | | | **2003-2010** | | | | **2011-2018** | | | |
| --- | --- | --- | --- | --- | --- | --- | --- | --- | --- | --- | --- | --- | --- |
|  |  | **NET** | | **NEC** | | **NET** | | **NEC** | | **NET** | | **NEC** | |
| Age (years, Median(IQR) | | 62(47-73) | | 68(58-76) | | 61(45-72) | | 68(58-76) | | 64(50-73) | | 70(60-78) | |
| Total | | **6192** | | **4423** | | **7589** | | **10306** | | **17862** | | **12204** | |
|  | | **n** | **%** | **n** | **%** | **n** | **%** | **n** | **%** | **n** | **%** | **n** | **%** |
| Sex | Male | 2960 | 47.8% | 2220 | 50.2% | 3547 | 46.7% | 5383 | 52.2% | 8201 | 45.9% | 6704 | 54.9% |
|  | Female | 3232 | 52.2% | 2203 | 49.8% | 4042 | 53.3% | 4923 | 47.8% | 9661 | 54.1% | 5500 | 45.1% |
| Ethnicity | Asian | 79 | 1.3% | 42 | 0.9% | 215 | 2.8% | 237 | 2.3% | 674 | 3.8% | 278 | 2.3% |
|  | Black | 55 | 0.9% | 39 | 0.9% | 124 | 1.6% | 148 | 1.4% | 444 | 2.5% | 180 | 1.5% |
|  | Mixed race | 11 | 0.2% | 5 | 0.1% | 34 | 0.4% | 36 | 0.3% | 94 | 0.5% | 40 | 0.3% |
|  | Other | 36 | 0.6% | 25 | 0.6% | 61 | 0.8% | 77 | 0.7% | 227 | 1.3% | 130 | 1.1% |
|  | White | 4202 | 67.9% | 2529 | 57.2% | 6863 | 90.4% | 9193 | 89.2% | 15522 | 86.9% | 11133 | 91.2% |
|  | Unknown | 1809 | 29.2% | 1783 | 40.3% | 292 | 3.8% | 615 | 6.0% | 901 | 5.0% | 443 | 3.6% |
| Site of tumour | Appendix | 1410 | 22.8% | 10 | 0.2% | 1919 | 25.3% | 185 | 1.8% | 3264 | 18.3% | 243 | 2.0% |
|  | Caecum | 255 | 4.1% | 52 | 1.2% | 231 | 3.0% | 196 | 1.9% | 476 | 2.7% | 201 | 1.6% |
|  | Colon | 185 | 3.0% | 82 | 1.9% | 185 | 2.4% | 238 | 2.3% | 293 | 1.6% | 404 | 3.3% |
|  | Lung | 1208 | 19.5% | 759 | 17.2% | 1782 | 23.5% | 2154 | 20.9% | 3829 | 21.4% | 2198 | 18.0% |
|  | Other | 1050 | 17.0% | 2845 | 64.3% | 1100 | 14.5% | 5063 | 49.1% | 1695 | 9.5% | 6289 | 51.5% |
|  | Pancreas | 223 | 3.6% | 364 | 8.2% | 209 | 2.8% | 1028 | 10.0% | 2117 | 11.9% | 1138 | 9.3% |
|  | Rectum | 277 | 4.5% | 86 | 1.9% | 344 | 4.5% | 332 | 3.2% | 1104 | 6.2% | 479 | 3.9% |
|  | Small intestine | 1300 | 21.0% | 79 | 1.8% | 1431 | 18.9% | 679 | 6.6% | 4132 | 23.1% | 714 | 5.9% |
|  | Stomach | 284 | 4.6% | 146 | 3.3% | 388 | 5.1% | 431 | 4.2% | 952 | 5.3% | 538 | 4.4% |
| Stage | Stage 1 | 22 | 0.4% | 28 | 0.6% | 155 | 2.0% | 134 | 1.3% | 4835 | 27.1% | 791 | 6.5% |
|  | Stage 2 | 11 | 0.2% | 29 | 0.7% | 56 | 0.7% | 147 | 1.4% | 1849 | 10.4% | 855 | 7.0% |
|  | Stage 3 | 18 | 0.3% | 44 | 1.0% | 48 | 0.6% | 250 | 2.4% | 2208 | 12.4% | 1112 | 9.1% |
|  | Stage 4 | 38 | 0.6% | 109 | 2.5% | 229 | 3.0% | 1016 | 9.9% | 2761 | 15.5% | 4211 | 34.5% |
|  | Unclassified | 6103 | 98.6% | 4213 | 95.3% | 7101 | 93.6% | 8759 | 85.0% | 6209 | 34.8% | 5235 | 42.9% |
| IMD | 1 – Least deprived | 1207 | 19.5% | 716 | 16.2% | 1528 | 20.1% | 1920 | 18.6% | 3756 | 21.0% | 2348 | 19.2% |
|  | 2 | 1336 | 21.6% | 879 | 19.9% | 1642 | 21.6% | 2097 | 20.3% | 3835 | 21.5% | 2565 | 21.0% |
|  | 3 | 1291 | 20.8% | 832 | 18.8% | 1612 | 21.2% | 2106 | 20.4% | 3798 | 21.3% | 2523 | 20.7% |
|  | 4 | 1206 | 19.5% | 956 | 21.6% | 1554 | 20.5% | 2001 | 19.4% | 3263 | 18.3% | 2394 | 19.6% |
|  | 5 – Most deprived | 1152 | 18.6% | 1040 | 23.5% | 1253 | 16.5% | 2182 | 21.2% | 3210 | 18.0% | 2374 | 19.5% |

**Supplementary Table 2**: (A) Demographics of 58,576 NET and NEC recorded on the NCRAS database between 1995-2018
