# Supplementary material for: Incidence and survival of neuroendocrine neoplasia in England 1995–2018: A retrospective, population-based study
Source: Lancet Reg Health Eur. 2022 Sep 23;23:100510. doi: 10.1016/j.lanepe.2022.100510 (PMC9513765; doi:10.1016/j.lanepe.2022.100510)
Supplement: Supplementary file 5 [file mmc5.docx]

| Site | Morphology | n | Median age (IQR) | Males% |
| --- | --- | --- | --- | --- |
| Appendix | NET | 6593 | 39(25-58) | 39.0% |
|  | NEC | 438 | 44.5 (25-63) | 44.1% |
| Caecum | NET | 962 | 67(58-74) | 45.1% |
|  | NEC | 449 | 70(61-77) | 44.8% |
| Colon | NET | 663 | 68(57-76) | 57.3% |
|  | NEC | 724 | 70(60-78) | 57.0% |
| Lung | NET | 6819 | 64(52-72) | 38.6% |
|  | NEC | 5111 | 68(60-75) | 52.5% |
| Pancreas | NET | 2549 | 63(51-71) | 52.6% |
|  | NEC | 2530 | 63(52-72) | 55.8% |
| Rectum | NET | 1725 | 56(48-68) | 51.9% |
|  | NEC | 897 | 68(56-77) | 63.1% |
| Small intestine | NET | 6863 | 68(59-76) | 57.1% |
|  | NEC | 1472 | 67(59-76) | 53.5% |
| Stomach | NET | 1624 | 67(56-76) | 43.8% |
|  | NEC | 1115 | 71(62-79) | 64.2% |

**Supplementary Table 2B**: Sex distribution of 40,534 primary site NET and NEC between 1995-2018
