# Supplementary material for: Incidence and survival of neuroendocrine neoplasia in England 1995–2018: A retrospective, population-based study
Source: Lancet Reg Health Eur. 2022 Sep 23;23:100510. doi: 10.1016/j.lanepe.2022.100510 (PMC9513765; doi:10.1016/j.lanepe.2022.100510)
Supplement: Supplementary file 6 [file mmc6.docx]

| **Site, tumours (Total = 14834)** | **Stage** | | | | | | | | | | | |
| --- | --- | --- | --- | --- | --- | --- | --- | --- | --- | --- | --- | --- |
|  | **1** | | | **2** | | | **3** | | | **4** | | |
| **Morphology** |  | **NET** | **NEC** |  | **NET** | **NEC** |  | **NET** | **NEC** |  | **NET** | **NEC** |
| **Appendix,** 2146 | 53% | 3% | 97% | 33% | 2% | 98% | 11% | 95% | 5% | 3% | 71% | 29% |
| **Caecum,** 528 | 2% | 85% | 15% | 6% | 70% | 30% | 41% | 78% | 22% | 51% | 65% | 35% |
| **Colon,** 509 | 9% | 89% | 11% | 6% | 32% | 68% | 22% | 38% | 62% | 63% | 30% | 70% |
| **Lung,** 4661 | 52% | 87% | 13% | 9% | 77% | 23% | 9% | 52% | 48% | 30% | 26% | 74% |
| **Pancreas,** 2183 | 23% | 89% | 11% | 18% | 91% | 9% | 10% | 79% | 21% | 49% | 54% | 46% |
| **Rectum,** 948 | 57% | 96% | 4% | 5% | 59% | 41% | 9% | 29% | 71% | 29% | 16% | 84% |
| **Small intestine,** 3201 | 7% | 98% | 2% | 9% | 96% | 4% | 40% | 96% | 4% | 44% | 86% | 14% |
| **Stomach,** 658 | 27% | 96% | 4% | 14% | 78% | 22% | 14% | 27% | 73% | 45% | 22% | 78% |

**Supplementary Table 3**: Stage of 14,834 main primary site NEN between 2012-2018 displaying proportion of NET and NEC in each subset
