# Supplementary material for: Incidence and survival of neuroendocrine neoplasia in England 1995–2018: A retrospective, population-based study
Source: Lancet Reg Health Eur. 2022 Sep 23;23:100510. doi: 10.1016/j.lanepe.2022.100510 (PMC9513765; doi:10.1016/j.lanepe.2022.100510)
Supplement: Supplementary file 7 [file mmc7.docx]

| **Stage** |  | **1** |  | **2** |  | **3** |  | **4** |  |
| --- | --- | --- | --- | --- | --- | --- | --- | --- | --- |
| **Site** | **YS** | **NET** | **NEC** | **NET** | **NEC** | **NET** | **NEC** | **NET** | **NEC** |
| **Appendix** | **1** | 98% | 94% | 97% | 100% | 100% | 73% | 76% | 44% |
|  | **3** | 95% | 94% | 94% | 100% | 96% | 64% | 49% | 11% |
|  | **5** | 93% | 82% | 90% | 100% | 95% | 55% | 42% | 11% |
| **Caecum** | **1** | 90% | 50% | 95% | 78% | 95% | 49% | 72% | 16% |
|  | **3** | 90% | 50% | 95% | 66% | 90% | 26% | 57% | 9% |
|  | **5** | 90% | 50% | 75% | 53% | 82% | 21% | 48% | 6% |
| **Colon** | **1** | 98% | 80% | 83% | 86% | 89% | 70% | 56% | 15% |
|  | **3** | 95% | 80% | 74% | 70% | 78% | 37% | 40% | 4% |
|  | **5** | 95% | 80% | 74% | 58% | 63% | 29% | 18% | 3% |
| **Lung** | **1** | 98% | 90% | 96% | 81% | 92% | 60% | 50% | 21% |
|  | **3** | 94% | 79% | 93% | 59% | 75% | 27% | 28% | 7% |
|  | **5** | 91% | 75% | 82% | 47% | 54% | 21% | 12% | 4% |
| **Pancreas** | **1** | 97% | 88% | 95% | 86% | 94% | 65% | 63% | 38% |
|  | **3** | 92% | 80% | 91% | 65% | 82% | 37% | 43% | 18% |
|  | **5** | 87% | 71% | 85% | 59% | 71% | 33% | 26% | 11% |
| **Rectum** | **1** | 99% | 95% | 96% | 79% | 88% | 63% | 55% | 15% |
|  | **3** | 98% | 80% | 96% | 42% | 77% | 29% | 28% | 2% |
|  | **5** | 97% | 74% | 87% | 29% | 65% | 19% | 22% | 1% |
| **Small intestine** | **1** | 96% | 80% | 93% | 82% | 94% | 80% | 78% | 55% |
|  | **3** | 89% | 80% | 83% | 62% | 88% | 67% | 64% | 43% |
|  | **5** | 86% | 60% | 73% | 44% | 80% | 58% | 43% | 32% |
| **Stomach** | **1** | 99% | 75% | 94% | 35% | 92% | 58% | 46% | 7% |
|  | **3** | 89% | 61% | 84% | 18% | 78% | 27% | 35% | 4% |
|  | **5** | 84% | 37% | 72% | 18% | 70% | 22% | 21% | 2% |

| Colour | % Survival |
| --- | --- |
|  | 75-100 |
|  | 50-74 |
|  | 25-49 |
|  | 0-24 |

**Supplementary Table 4.** Kaplan-Meier predicted 1–, 3– and 5–year overall survival of 14,834 NEN between 2012–2018 in England. YS= K-M Predicted survival (years). NET = Neuroendocrine tumour. NEC = Neuroendocrine carcinoma. *Note: this table does not adjust for age, sex or other variables, it should be used only as a reference for clinicians, not as a prognostic tool.*
